# Self‐Esteem and Repetitive Negative Thinking as Mediators of the Association Between Bullying and Disordered Eating

**DOI:** 10.1002/jclp.70186

**Published:** 2026-07-22

**Authors:** Kaitlynn E. Ellis, Camille Archer, Gabrielle E. Reimann, Yulan D. Chen, Ralph E. Francois, Antonia N. Kaczkurkin

**Affiliations:** ^1^ Department of Psychology Vanderbilt University Nashville Tennessee USA

**Keywords:** bullying, eating disorder symptoms, emerging adults, repetitive negative thinking, self‐esteem, young adults

## Abstract

**Objective:**

Eating disorders are associated with marked distress and impairment, including elevated risk for anxiety, depression, and suicidality. Bullying victimization is associated with eating disorder symptoms in late adolescence and young adulthood, when peer relationships and social evaluation are developmentally salient. Cognitive‐affective processes, including self‐esteem and repetitive negative thinking, may help explain this association; however, less is known about whether these constructs show unique indirect associations when examined simultaneously.

**Methods:**

Self‐esteem and repetitive negative thinking were examined as parallel statistical mediators of the association between bullying victimization and eating disorder symptoms in a large community sample of emerging and young adults aged 17–23 years (*N* = 2132). Participants completed validated self‐report measures of bullying victimization, eating disorder symptoms, self‐esteem, and repetitive negative thinking.

**Results:**

Greater bullying victimization was associated with higher eating disorder symptoms, lower self‐esteem, and greater repetitive negative thinking. In the parallel mediation model, both self‐esteem and repetitive negative thinking showed significant indirect effects. The direct effect of bullying victimization remained significant, indicating partial mediation. The indirect effect through self‐esteem was significantly larger than the indirect effect through repetitive negative thinking.

**Conclusion:**

These findings suggest that self‐esteem and repetitive negative thinking are distinct cognitive‐affective correlates of the association between bullying victimization and eating disorder symptoms in emerging and young adults. Although the indirect effect through self‐esteem was larger, both processes may be clinically relevant. Findings highlight the potential value of assessing bullying‐related distress, self‐evaluative concerns, and repetitive negative thinking among individuals with elevated eating disorder symptoms.

## 1 Introduction

Eating disorders are complex mental health conditions characterized by persistent disturbances in eating behavior that compromise physical health and psychosocial functioning (American Psychiatric Association 2022; Lipson and Sonneville 2017; Micali et al. 2014; Tomba et al. 2019; Walsh 2011). Eating disorders are prevalent across the lifespan, with estimates ranging from 3.3% to 18.6% in women and 0.8% to 6.5% in men (Galmiche et al. 2019; Smink et al. 2012). Beyond their immediate effects on eating behavior and self‐image, these disorders are associated with severe long‐term consequences, including social isolation, heightened risk for comorbid psychopathology such as depression, anxiety, and substance use disorders, and increased mortality (Agras 2001; Smink et al. 2012). Given this substantial burden, it is critical to identify the factors that contribute to the maintenance of eating disorder symptoms.

One psychosocial factor that has received increasing attention in eating disorder research is the role of peer relationships. Emerging and young adulthood, spanning late adolescence into the 20s, represents a critical time for social identity formation and increased independence from the family, during which peer influence may become particularly salient (Giletta et al. 2021). As emerging and young adults begin to rely more heavily on peers for social support, peer dynamics may shape norms around body image and dieting, reinforcing disordered eating behaviors and social pressures related to weight and shape (Lukas et al. 2022; Meyer and Gast 2008; Quiles Marcos et al. 2013; Shomaker and Furman 2009). Peer victimization, including bullying, has emerged as a significant risk factor for eating disorder symptoms (Cook‐Cottone et al. 2016; Copeland et al. 2015; de Oliveira Galvão et al. 2023; Lie et al. 2019; Marco et al. 2018; Oliveira et al. 2023). However, the pathways through which bullying contributes to eating disorder symptoms remain unclear.

Cognitive‐affective factors, such as self‐esteem and repetitive negative thinking, may help clarify the relationship between peer victimization and eating pathology, as both have been associated with the development and maintenance of disordered eating behaviors (Culbert et al. 2015; McCabe and Vincent 2003; Serpell and Troop 2003). Both self‐esteem and repetitive negative thinking undergo developmental change and remain sensitive to social influence during late adolescence and into young adulthood (Wang et al. 2018; Watkins and Roberts 2020). During this period, the heightened salience of social evaluation, identity formation, and autonomy from family systems may make peer victimization particularly relevant for self‐evaluative and emotion‐regulatory processes. Bullying experiences may contribute to disordered eating not only through exposure to peer adversity but also through their association with lower self‐worth and more persistent negative self‐focused thinking.

Self‐esteem, an individual's evaluation of self‐worth and confidence, has been strongly implicated in eating disorder pathology. Low self‐esteem is consistently associated with elevated eating disorder symptoms (Beckers et al. 2023; Geller et al. 2002; Krauss et al. 2023; Mora et al. 2017; Zamani Sani et al. 2020), as self‐esteem may become enmeshed with negative perceptions of weight and body shape (Serpell and Troop 2003). Notably, experiences of bullying are also associated with self‐esteem (Tsaousis 2016), suggesting a potential pathway through which peer victimization may contribute to disordered eating. Although previous research has identified self‐esteem as a mediator in the relationship between bullying and eating disorder symptoms, self‐esteem does not fully account for the variance in eating disorder symptoms (Lee et al. 2017), indicating that additional processes may play a role. Cognitive‐behavioral theories of eating disorders posit that cognitive‐affective processes, such as repetitive negative thinking, may reinforce negative schemas derived from past experiences, thereby perpetuating eating disorder symptoms (Fox and Power 2009). Thus, repetitive negative thinking may be important in the development of eating pathology.

Repetitive negative thinking, a maladaptive cognitive process characterized by persistent focus on negative thoughts, adverse experiences, and negative emotions, has been implicated in multiple mental health conditions that commonly co‐occur with eating disorders, including depression and anxiety (Chu et al. 2019; Feinstein et al. 2014; Labella et al. 2024; Monti et al. 2017; Smith et al. 2018; Zou et al. 2023). Moreover, individuals with higher levels of repetitive negative thinking tend to exhibit more severe eating disorder symptoms (Cowdrey and Park 2012; Dondzilo et al. 2016; Palmieri et al. 2021; Rawal et al. 2010; Smith et al. 2018). Repetitive negative thinking may serve as a transdiagnostic process through which interpersonal stressors such as bullying may exacerbate internalizing symptoms and contribute to the maintenance of disordered eating (Parris et al. 2022; Smith et al. 2018). Given its associations with emotional dysregulation and maladaptive coping (Hong 2007; Zawadzki 2015), repetitive negative thinking may play an important role in the relationship between peer victimization and eating pathology. Despite these established associations, additional research is needed to examine whether repetitive negative thinking mediates the relationship between bullying and eating disorder symptoms.

Taken together, developmental and cognitive‐affective perspectives suggest that bullying may be associated with eating disorder symptoms through self‐evaluative processes and repetitive negative thinking; however, less is known about whether these factors uniquely account for the bullying–eating disorder association when examined simultaneously. The present study addresses this question by modeling self‐esteem and repetitive negative thinking as parallel statistical mediators, allowing examination of whether each factor shows a unique indirect association between bullying and eating disorder symptoms. We focused on individuals aged 17 to 23 years to capture the transition from late adolescence into young adulthood, when peer evaluation may be especially relevant to self‐esteem, repetitive negative thinking, and eating disorder symptoms. We hypothesized that individuals who experience recent bullying victimization would report lower self‐esteem, higher levels of repetitive negative thinking, and greater eating disorder symptoms. Both self‐esteem and repetitive negative thinking were expected to show significant indirect associations in the relationship between bullying and eating pathology.

## 2 Methods

### 2.1 Participants

This sample was drawn from a larger parent study (Durham et al. 2025) that recruited a community‐based cohort of individuals aged 12 years and older residing in a metropolitan area in the southern United States. The sample was restricted to individuals aged 17 to 23 years, as there were too few participants outside this age range to conduct reliable analyses. Of the full sample size of 2470, 198 participants were excluded for age, and 140 participants were excluded for missing data on the variables of interest. The final sample for analyses included 2132 participants (age: *M* = 19.83, SD = 1.38). The sample demographic characteristics are presented in Table 1. Using the established cutoff of ≥15 on the Eating Disorder Examination‐Questionnaire Short Form to indicate a probable eating disorder (Prnjak et al. 2020), 23.31% of the sample reported clinically significant eating disorder symptoms.

**Table 1:** Summary of the demographic characteristics of the sample (*N* = 2132).

	Mean	SD
Age (years)	19.83	1.38

	*N*	*%*
Sex		
Male	617	28.94
Female	1514	71.01
Missing	1	0.05
Race/Ethnicity		
American Indian or Alaskan Native	9	0.42
Asian	581	27.25
Black or African American	240	11.26
Hispanic	228	10.69
Native Hawaiian or Other Pacific Islander	8	0.38
White	946	44.37
Multiracial	109	5.11
Other	11	0.52
Household Annual Income		
$31,000 or less	144	6.75
$31,001–$42,000	177	8.30
$42,001–$126,000	701	32.88
$126,001–$188,000	405	19.00
$188,001 or more	705	33.07
Education		
Currently enrolled in high school	12	0.56
Didn't finish high school, but completed a technical or vocational program	1	0.05
High school graduate or GED	318	14.92
Completed high school and a technical or vocational program	18	0.84
Less than 2 years of college	1033	48.45
2 years of college or more, including associate degree or equivalent	376	17.64
College graduate (4‐ or 5‐year program)	330	15.48
Completed or enrolled in a master's degree or other post‐graduate training	31	1.45
Completed or enrolled in a doctoral degree program (PhD, MD, EdD, DVM, DDS, JD, etc.)	13	0.61

Abbreviations: DDS, Doctor of Dental Surgery; DVM, Doctor of Veterinary Medicine; EdD, Doctor of Education; GED, general education development/general education diploma; JD, Juris Doctor; MD, Doctor of Medicine; PhD, Doctor of Philosophy; SD, standard deviation.

### 2.2 Procedures

Participants were recruited through multiple community outreach methods, including targeted social media advertisements, postings on research‐focused websites, and physical flyers distributed throughout the greater Nashville area. Recruitment materials were intended to reach individuals in the general community rather than individuals seeking eating disorder treatment specifically. Flyers were distributed in public and community‐facing locations, and online advertisements directed interested individuals to complete an eligibility form for the larger parent study. Thus, the sample reflects a community‐based, nonclinical cohort recruited through both online and in‐person outreach. To be eligible for participation, individuals were required to be 12 years of age or older and proficient in English. Potential participants completed an interest form, and if they met the study's age and English proficiency requirements, they were sent a link to the online consent form. Informed consent was obtained from adult participants prior to survey completion. For participants aged 17 years or younger, assent was obtained from minors, and informed consent was obtained from caregivers. Participants were then directed to the online survey, which took approximately 1 h to complete. All questionnaires were administered using REDCap, a secure, web‐based platform designed for data collection and management in compliance with HIPAA regulations (Harris et al. 2009, 2019).

### 2.3 Measures

#### 2.3.1 Eating Disorder Examination—Questionnaire Short Form

The Eating Disorder Examination–Questionnaire Short Form (EDE‐QS) was used to measure symptoms related to eating pathology. The EDE‐QS is a 12‐item self‐report measure derived from the original 28‐item Eating Disorder Examination–Questionnaire (EDE‐Q) (Gideon et al. 2016). Respondents are asked to indicate how many of the past 7 days they engaged in disordered eating behaviors on a 4‐point Likert scale ranging from 0 (*0 days*) to 3 (*6*–*7 days*). Additionally, the measure includes items assessing the extent to which eating, weight, and shape have influenced self‐perception over the past 7 days, using a 4‐point Likert scale from 0 (*Not at all*) to 3 (*Markedly*). Consistent with standard EDE‐QS scoring procedures (Gideon et al. 2016), items were summed to create a total symptom severity score. Because all EDE‐QS items are scored on the same 0–3 scale, internal consistency was calculated using all 12 scored items. The EDE‐QS has shown high internal consistency in prior studies (*α* = 0.91) and is strongly associated with the original EDE‐Q and other measures of eating disorder symptoms (*r* = 0.61–0.82), including the Clinical Impairment Assessment (CIA) and Short Evaluation of Eating Disorders (SEED) (Gideon et al. 2016). In the current sample, the EDE‐QS displayed excellent internal consistency (*α* = 0.91).

#### 2.3.2 Forms of Bullying Scale—Victimization

Bullying experiences were assessed using the Forms of Bullying Scale–Victimization version (FBS‐V), which assesses recent bullying victimization. The FBS‐V measures five broad forms of bullying that can occur both online and offline: verbal (teasing, name‐calling), threatening (intimidation), physical (harm to individual or property), relational (damage to relationships), and social (spreading lies or rumors). Participants are asked to indicate how often they experienced different forms of bullying over the previous 6 months. Responses are rated on a 5‐point Likert scale from 0 (*This did not happen to me*) to 4 (*Several times a week or more*), with higher scores indicating more frequent exposure to bullying. The FBS‐V has shown high internal consistency (*α* = 0.87) in prior validation studies (Shaw et al. 2013) and in the current sample (*α* = 0.89).

#### 2.3.3 Rosenberg Self‐Esteem Scale

Self‐esteem was assessed using the Rosenberg Self‐Esteem Scale (RSES), a 10‐item self‐report questionnaire that measures positive and negative feelings and beliefs about oneself. Participants are asked to indicate how strongly they agree or disagree with statements such as “I feel that I'm a person of worth, at least on an equal plane with others,” using a 4‐point Likert scale ranging from 0 (*Strongly disagree*) to 3 (*Strongly agree*). Higher scores indicate higher self‐esteem (Rosenberg 1965). The Rosenberg Self‐Esteem Scale has demonstrated good internal consistency (*α* = 0.88) in prior studies (Fleming and Courtney 1984), which was also observed in the current sample (*α* = 0.90).

#### 2.3.4 Perseverative Thinking Questionnaire

Repetitive negative thinking was assessed using the Perseverative Thinking Questionnaire (PTQ). The PTQ is a 15‐item self‐report measure that evaluates repetitive negative thinking by asking participants to rate how they typically think about negative experiences or problems. For example, participants rate items such as, “The same thoughts keep going through my mind again and again.” Items are measured on a 5‐point Likert scale ranging from 0 (*Never*) to 4 (*Almost always*), with higher scores indicating greater repetitive negative thinking. The PTQ has shown excellent internal consistency (*α* = 0.94–0.95) in previous validation work (Ehring et al. 2011) and in the current sample (*α* = 0.96).

### 2.4 Data Analyses

All statistical analyses were performed using R in RStudio. Correlations among eating disorder symptoms, bullying experiences, self‐esteem, and repetitive negative thinking were computed. Prior to conducting inferential analyses, we examined the distributions of all key variables. The bullying variable was positively skewed and showed a substantial floor effect, thus, indirect effects were estimated using bootstrapped confidence intervals, which do not assume normality of the indirect effect. Additionally, influence diagnostics did not indicate that the results were driven by highly influential observations: the Cook's distance values were less than or equal to 0.078 across the mediator and outcome models, with no observations exceeding conventional thresholds of 0.50 or 1.00. As a sensitivity analysis, we tested our models with and without log‐transforming the bullying measure, as described below.

As reported in the results, total bullying victimization scores and eating disorder symptoms were significantly associated with age. To account for this association, age was included as a covariate in the subsequent mediation models. Because missing data on the primary analysis variables were relatively low (6.2% of the age‐eligible sample), complete‐case analyses were used for the primary models. We tested a parallel multiple mediation model using structural equation modeling to evaluate whether self‐esteem and repetitive negative thinking statistically mediated the association between bullying victimization and eating disorder symptoms. Both mediators were included simultaneously, allowing each indirect effect to be estimated while adjusting for the other mediator. Variance inflation factors were low across all predictors of interest, ranging from 1.01 to 1.49, suggesting that multicollinearity was not a substantial concern. Indirect, direct, and total effects were obtained via nonparametric bootstrapping with 5,000 bootstrap resamples, and standardized effects were computed by repeating the analyses on standardized variables. The proportion mediated was calculated as the ratio of the indirect effect to the total effect and was estimated as a bootstrap‐defined parameter within the structural equation model. To compare the relative magnitude of the two indirect effects, we estimated a contrast representing the difference between the indirect effect through self‐esteem and the indirect effect through repetitive negative thinking and derived bootstrap confidence intervals for this contrast.

Finally, we conducted sensitivity analyses to examine the robustness of the results. First, because self‐esteem and repetitive negative thinking were moderately correlated, we estimated separate models in which each variable was tested individually as a mediator of the association between bullying and eating disorder symptoms. Second, given the skewed distribution of bullying scores, we repeated the analyses with a log‐transformed bullying variable to evaluate whether the pattern of findings was robust to this alternative model specification. Third, to evaluate the robustness of the findings to the handling of missing data, we conducted a sensitivity analysis using multiple imputation by chained equations with predictive mean matching (20 imputations). The primary parallel mediation model was re‐estimated in each imputed dataset, and pathway coefficients, indirect effects, direct effects, and total effects were pooled across imputations using Rubin's rules. Analysis code is available at https://github.com/VU-BRAINS-lab/Ellis_Bullying_Eating.

## 3 Results

### 3.1 Correlations Between Eating Disorder Symptoms, Bullying, Self‐Esteem, and Repetitive Negative Thinking

Bivariate Pearson correlations among the primary study variables are presented in Table 2. Greater exposure to bullying in the past 6 months was significantly correlated with higher levels of eating disorder symptoms over the past week. Bullying exposure and eating disorder symptoms were both significantly associated with elevated repetitive negative thinking and lower self‐esteem. In addition, repetitive negative thinking and self‐esteem were significantly correlated with each other, such that greater repetitive negative thinking was related to lower self‐esteem.

**Table 2:** Correlations between measures of interest.

Variable	*M*	SD	1	2	3	4
1. Eating disorder symptoms	8.72	7.84	—	—	—	—
2. Bullying	13.06	4.69	0.27***	—	—	—
3. Repetitive negative thinking	27.40	13.16	0.31***	0.21***	—	—
4. Self‐esteem	19.18	5.43	−0.38***	−0.22***	−0.57***	—

Abbreviations: M, mean; SD, standard deviation.

*** *p* < 0.001.

### 3.2 Relationship Between Bullying and Age

Regression models examining the associations between age and each primary study variable showed that total bullying scores were significantly associated with age (Table S1), with younger individuals reporting more bullying experiences. Age was also related to eating disorder symptom severity, such that symptom levels were higher among younger participants. Self‐esteem and repetitive negative thinking were not significantly associated with age. To account for age‐related differences in bullying victimization and eating disorder symptoms, age was included as a covariate in subsequent analyses.

### 3.3 Self‐Esteem and Repetitive Negative Thinking as Mediators of the Association Between Bullying and Eating Disorder Symptoms

**Figure 1:**
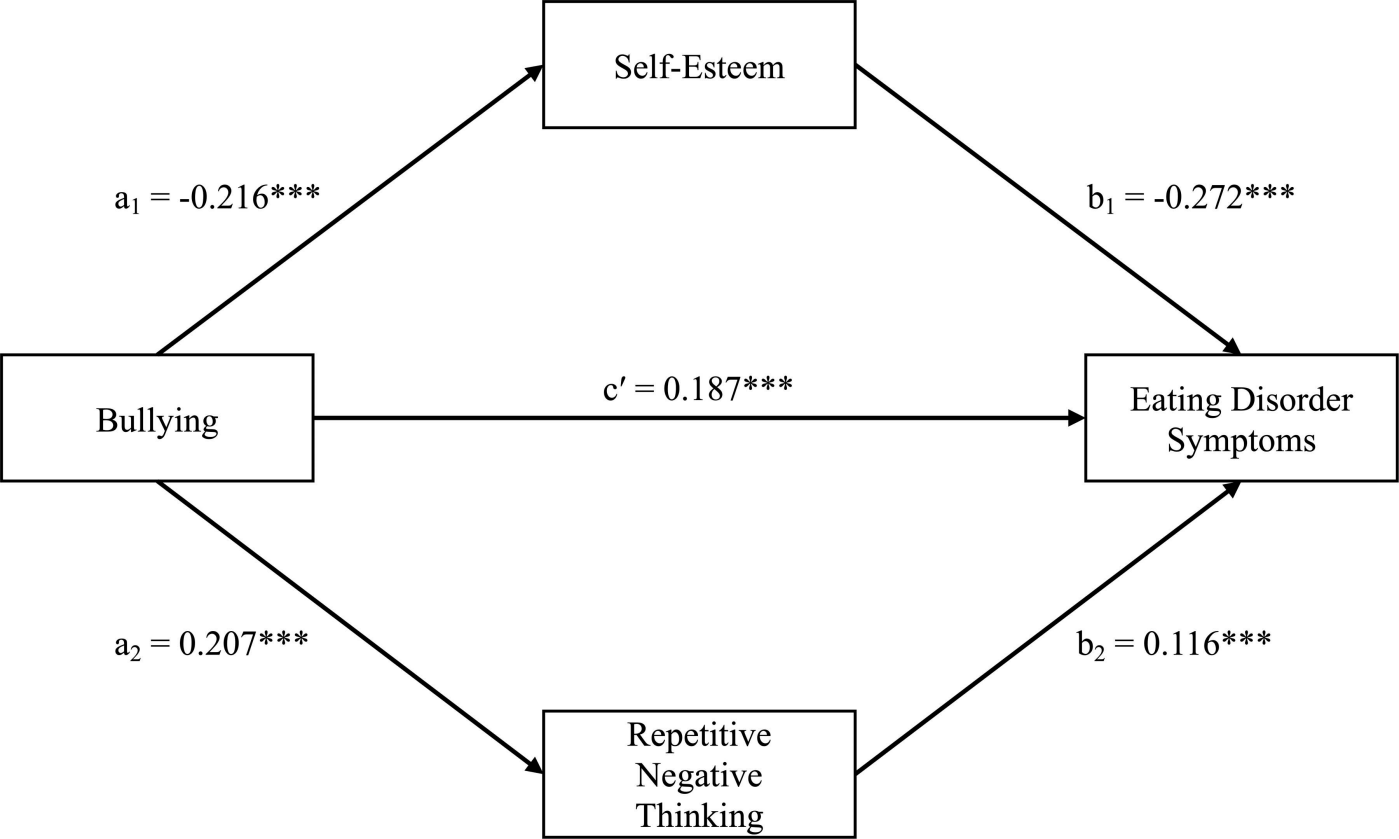
Self‐esteem and repetitive negative thinking as parallel mediators of the association between bullying victimization and eating disorder symptoms. Standardized coefficients are shown. Age was included as a covariate in the mediator and outcome models. Self‐esteem and repetitive negative thinking were modeled simultaneously as parallel mediators. c’ represents the direct effect of bullying on eating disorder symptoms after accounting for both mediators. ***p < 0.001.

Standardized estimates, standard errors, 95% bootstrap confidence intervals, and *p*‐values for the mediation analyses are presented in Table 3, and the mediation model is shown in Figure 1. Exposure to bullying in the past 6 months was positively associated with eating disorder symptoms during the past week (total effect, *p* < 0.001). In terms of the self‐esteem pathway, higher bullying exposure was associated with lower self‐esteem (a_1_ path, *p* < 0.001), and lower self‐esteem was related to greater eating disorder symptoms after adjusting for bullying and repetitive negative thinking (b_1_ path, *p* < 0.001). The indirect effect via self‐esteem was significant (*p* < 0.001), accounting for 21.77% (95% bootstrap CI [16.29%, 28.88%]) of the total effect, and the direct effect remained significant (*p* < 0.001). In parallel, bullying was associated with greater repetitive negative thinking (a_2_ path, *p* < 0.001), and repetitive negative thinking was associated with higher eating disorder symptoms after adjusting for bullying and self‐esteem (b_2_ path, *p* < 0.001). The indirect effect through repetitive negative thinking was also significant (*p* < 0.001), representing 8.87% of the total effect (95% bootstrap CI: [4.84%, 13.63%]). Standardized estimates indicated small indirect effects through self‐esteem (*β* = 0.059) and repetitive negative thinking (*β* = 0.024), alongside a modest direct effect (*β* = 0.187). The indirect effect through self‐esteem was significantly larger than the indirect effect through repetitive negative thinking, as indicated by a significant contrast between the two indirect effects (*β* = 0.035, 95% bootstrapped CI: [0.016, 0.056], *p* = 0.001).

**Table 3:** Self‐esteem and repetitive negative thinking as parallel mediators of the association between bullying and eating disorder symptoms.

Self‐Esteem^								
				95% Bootstrapped CI				
Type	Effect	*β*	SE	Lower	Upper	*z*	*p*	% Mediated [95% CI]
Indirect	Bullying → Self‐esteem → Eating disorder symptoms	0.059	0.008	0.045	0.075	7.67	< 0.001	21.77 [16.29, 28.88]
a path	Bullying → Self‐esteem	−0.216	0.020	−0.257	−0.178	−10.45	< 0.001	—
b path	Self‐esteem → Eating disorder symptoms	−0.272	0.024	−0.320	−0.225	−11.19	< 0.001	—

**Repetitive Negative Thinking^**								
				95% Bootstrapped CI				
Type	Effect	*β*	SE	Lower	Upper	*z*	*p*	% Mediated [95% CI]
Indirect	Bullying → Repetitive negative thinking → Eating disorder symptoms	0.024	0.006	0.013	0.036	4.12	< 0.001	8.87 [4.84, 13.63]
a path	Bullying → Repetitive negative thinking	0.207	0.023	0.161	0.254	8.87	< 0.001	—
b path	Repetitive negative thinking → Eating disorder symptoms	0.116	0.025	0.066	0.165	4.59	< 0.001	—
Direct	Bullying → Eating disorder symptoms	0.187	0.026	0.138	0.240	7.22	< 0.001	—
Total	Bullying → Eating disorder symptoms	0.270	0.027	0.218	0.324	9.90	< 0.001	—

*Note:* ^Each model controls for the other mediator.

Abbreviations: β, standardized coefficient; CI, confidence interval; *p,* *p*‐value; SE, standard error.

### 3.4 Sensitivity Analyses

Given the moderate to strong correlation between self‐esteem and repetitive negative thinking, we examined whether the indirect effects were retained when each mediator was modeled separately. The pattern of results was consistent with the main parallel mediation model, with both self‐esteem and repetitive negative thinking showing significant indirect effects when tested separately (Table S2). We also repeated the mediation model using a log‐transformed bullying variable to address the positive skew and floor effect of bullying scores. The direction and magnitude of the effects were consistent with the primary analyses: bullying remained indirectly associated with eating disorder symptoms through both self‐esteem and repetitive negative thinking, and the direct effect of bullying on eating disorder symptoms remained significant (Table S3). The findings were also consistent when missing data were handled using multiple imputation, indicating that the results were robust to the approach used to handle missing data (Table S4).

## 4 Discussion

The current study aimed to extend prior research on bullying experiences and eating disorder symptoms by assessing the roles of self‐esteem and repetitive negative thinking as statistical mediators. Consistent with prior work, bullying was associated with greater eating disorder symptoms, lower self‐esteem, and increased repetitive negative thinking. Mediation analyses further indicated significant indirect effects through both self‐esteem and repetitive negative thinking in the association between bullying and eating pathology. The indirect effect through self‐esteem was significantly larger than the indirect effect through repetitive negative thinking in the present model. These findings suggest that self‐esteem and repetitive negative thinking may be important cognitive‐affective processes in understanding the relationship between bullying experiences and disordered eating, while also underscoring that each construct may capture distinct aspects of this relationship.

The present results align closely with prior research showing an association between peer victimization and eating disorder symptoms (Copeland et al. 2015; Day et al. 2022; Lie et al. 2019; Oliveira et al. 2023). These results are also consistent with studies identifying self‐esteem as a mediator of the association between bullying and eating disorder symptoms (Duarte et al. 2015; Giletta et al. 2010; Lee et al. 2017), suggesting that self‐evaluation may be one process through which peer victimization could be associated with disordered eating. The current study extends this work by examining repetitive negative thinking alongside self‐esteem, allowing evaluation of whether each construct showed a unique indirect association with eating disorder symptoms when modeled simultaneously. In this model, the indirect effect through self‐esteem was significantly larger than the indirect effect through repetitive negative thinking, indicating that global self‐worth may be especially relevant in this sample. However, this finding should not be interpreted to suggest that self‐esteem is the only correlate, or necessarily the most clinically important correlate, of the relationship between bullying and eating pathology. For example, a prior longitudinal analysis found that self‐esteem did not mediate the association between interpersonal peer problems and disordered eating behaviors 2 years later, despite remaining highly correlated with disordered eating behaviors (Beckers et al. 2023). Consistent with our finding of partial mediation, this prior work suggests that self‐esteem alone does not fully account for the association between peer adversity and eating pathology. Thus, although self‐esteem appears to be an important factor, other cognitive‐affective processes likely contribute to eating disorder symptoms following bullying experiences.

One reason self‐esteem may have shown a larger indirect effect in the current study is that low self‐esteem is a transdiagnostic vulnerability factor that may amplify the effects of social rejection, perceived inferiority, and appearance‐based comparison (Kresznerits et al. 2022). For individuals exposed to bullying, prior work suggests that negative self‐concept may become intertwined with broader self‐evaluative concerns relevant to eating disorder symptoms, including body image concerns (Geller et al. 2002; Krauss et al. 2023; Mora et al. 2017; Zamani Sani et al. 2020), possibly as a means of regaining perceived control or social approval (Day et al. 2022). This may be especially salient in emerging and young adulthood, when increasing reliance on peers coincides with identity consolidation and pronounced body image concerns (Potterton et al. 2020). However, because we did not assess body image concerns, internalized weight bias, perceived control, or motives related to social approval, we cannot draw inferences about such processes; these potential factors should be examined directly in future research.

Although the indirect effect through repetitive negative thinking was smaller than the indirect effect through self‐esteem, the role of repetitive negative thinking as a mediator provides additional insight into the cognitive processes associated with peer victimization and eating pathology. Individuals who engage in repetitive negative thinking about past social experiences may sustain emotional distress following bullying experiences (Barchia and Bussey 2010; Feinstein et al. 2014), potentially contributing to body dissatisfaction and maladaptive coping strategies, such as restrictive eating or binge‐purge cycles (Cowdrey and Park 2012; Dondzilo et al. 2016; Naumann et al. 2015; Palmieri et al. 2021; Rawal et al. 2010). This pattern is consistent with prior work showing that repetitive negative thinking mediates associations between bullying and other forms of internalizing psychopathology, including depression and suicidal ideation (Chu et al. 2019; Feinstein et al. 2014; Monti et al. 2017; Zou et al. 2023). Extending these models to eating disorder symptoms highlights repetitive negative thinking as a potential transdiagnostic process that may be associated with both interpersonal stress and maladaptive behavioral outcomes. Importantly, the smaller indirect effect observed for repetitive negative thinking in the present model does not diminish its potential relevance; rather, it suggests that repetitive negative thinking may operate alongside self‐esteem and other cognitive‐affective processes in the relationship between bullying and eating disorder symptoms. Future longitudinal studies are needed to clarify the temporal dynamics of this association, including whether repetitive negative thinking serves as a factor that prolongs eating disorder symptoms over time in those who experience bullying.

Our findings are broadly consistent with the theoretical model of interpersonal psychotherapy for eating disorders (IPT‐ED), which posits a reciprocal process whereby negative social evaluation, low self‐esteem, and negative affect increase vulnerability to eating disorder symptoms and maintain interpersonal difficulties over time (Rieger et al. 2010). For those with a history of bullying victimization, low self‐esteem and increased repetitive negative thinking may be associated with more severe disordered eating symptoms and may help maintain this maladaptive cycle. For example, Trauma‐Focused Cognitive Behavioral Therapy for Weight Bullying (TF‐CBT‐WB) conceptualizes bullying as a potentially traumatic interpersonal experience and directly addresses bullying‐related distress in eating disorder treatment (Lydecker et al. 2024). Preliminary findings suggest that this approach may be associated with reductions in eating disorder symptoms (Lydecker et al. 2024). However, given the cross‐sectional and community‐based nature of the present study, our findings should not be interpreted as identifying treatment targets or informing clinical practice directly. Future longitudinal and intervention studies are needed to determine whether changes in self‐esteem and repetitive negative thinking are prospectively associated with eating disorder symptom trajectories or treatment response among individuals with histories of bullying victimization.

This study has several strengths. First, the use of a large, community‐based sample of emerging and young adults allowed us to examine bullying, self‐esteem, repetitive negative thinking, and eating disorder symptoms during a developmental period in which peer relationships, self‐evaluation, and cognitive‐affective processes remain highly salient. Second, the inclusion of repetitive negative thinking extends prior work on the bullying–eating disorder relationship, which has more commonly focused on self‐esteem, by examining whether repetitive negative thinking also contributes to this association. Finally, the use of validated measures strengthens confidence in the assessment of the primary constructs. Nevertheless, several limitations warrant consideration.

The cross‐sectional design of this study precludes causal inference. Accordingly, the present findings should be interpreted as evidence of statistical mediation rather than causal mediation. Causal mediation would require assumptions that cannot be fully evaluated in the present design, including temporal ordering of bullying, self‐esteem, repetitive negative thinking, and eating disorder symptoms, as well as the absence of unmeasured confounding among the predictor, mediator, and outcome variables. Because all variables were assessed at a single time point, longitudinal or experimental designs are needed to determine whether bullying leads to subsequent changes in these factors over time. In addition, the measures used in this study assessed constructs across different reference periods. For example, the bullying measure assessed victimization experiences within the past 6 months and did not capture childhood or lifetime exposure to bullying, whereas eating disorder symptoms were assessed over the previous 7 days. In contrast, the measures of self‐esteem and repetitive negative thinking assessed more trait‐like tendencies, asking participants to report how they typically think or feel across a range of situations. These differences in assessment timeframes complicate interpretation of the observed indirect associations, as the constructs were not measured over comparable periods. Consequently, the present findings cannot address whether earlier bullying experiences contribute to the development or progression of eating disorder symptoms over time. Reliance on self‐report measures also introduces potential recall and social desirability biases. Future research would benefit from incorporating multi‐method assessments, such as clinician interviews, behavioral tasks, or ecological momentary assessment.

In addition, the present study focused on global self‐esteem and general repetitive negative thinking rather than body image‐specific constructs, such as body esteem, weight/shape overvaluation, internalized weight bias, or preoccupation with weight, shape, and eating. These domain‐specific constructs may be more proximally related to eating disorder symptoms and may capture processes not fully represented by global self‐esteem or general repetitive negative thinking. Future research should examine whether validated, multi‐item measures of these body image‐specific constructs account for greater variance in the relationship between bullying experiences and eating pathology. Relatedly, although self‐esteem and repetitive negative thinking showed significant indirect effects, the indirect effect sizes were modest, which is important given the large sample size and increased likelihood of detecting statistically significant effects. Thus, the magnitude of these effects should be interpreted cautiously as additional processes likely contribute to the association between bullying and disordered eating. Prior research has identified other factors that may help account for the association between bullying and disordered eating, including body dissatisfaction, shame, and anxiety, which may function as additional mediators (Bellows et al. 2023; Cook‐Cottone et al. 2016; Xie et al. 2024). Future research should examine how these and other psychological and social processes may operate alongside self‐esteem and repetitive negative thinking to shape eating pathology.

Furthermore, the developmental heterogeneity of the sample should be considered when interpreting the findings. Although the 17–23 age range captures a meaningful transition from late adolescence to young adulthood, this period includes individuals who may differ substantially in dependence status, living situation, educational context, autonomy, and exposure to peer environments. These differences may influence both opportunities for bullying victimization and the ways in which bullying is associated with self‐esteem, repetitive negative thinking, and eating disorder symptoms. Future research should examine whether these associations vary across narrower developmental subgroups, such as children, adolescents, transition‐age youth, and young adults. Finally, while our sample was moderately diverse, replication across cultural contexts and gender identities would help clarify the generalizability of these findings.

In summary, the current study showed that both self‐esteem and repetitive negative thinking statistically mediated the association between bullying and eating disorder symptoms in emerging and young adults, with a larger indirect effect observed through self‐esteem in the present model. These results underscore the importance of considering both self‐evaluative and cognitive processes in future research on assessments and interventions focused on the psychological distress associated with peer victimization. Collectively, these findings contribute to a growing understanding of how social adversity is associated with cognitive and affective processes that may contribute to eating disorder symptoms.

## Author Contributions

**Kaitlynn E. Ellis:** conceptualization, formal analysis, investigation, writing – original draft, writing – review and editing. **Camille Archer:** investigation, writing – review and editing. **Gabrielle E. Reimann:** investigation, writing – review and editing. **Yulan D. Chen:** investigation, writing – review and editing. **Ralph E. Francois:** investigation, writing – review and editing. **Antonia N. Kaczkurkin:** conceptualization, formal analysis, methodology, writing – review and editing, funding acquisition, resources, supervision.

## Conflicts of Interest

The authors declare no conflicts of interest.

## IRB Statement

We complied with the APA's ethical standards regarding the collection and use of human subjects’ data. The study procedures received approval from the Vanderbilt University Institutional Review Board (IRB #192334).

## Supporting information


Supporting File

## Data Availability

All R code for these analyses can be found at https://github.com/VU-BRAINS-lab/Ellis_Bullying_Eating. Data can be obtained by contacting the corresponding author. The study was not preregistered. We report all data exclusions, all manipulations, and all measures in the study.
