# Supplementary material for: Self‐Esteem and Repetitive Negative Thinking as Mediators of the Association Between Bullying and Disordered Eating
Source: J Clin Psychol. 2026 Jul 22;82(10):1617–27. doi: 10.1002/jclp.70186 (PMC13544460; doi:10.1002/jclp.70186)
Supplement: Supplementary file 1 [file JCLP-82-1617-s001.docx]

Supplementary materials for “Self-Esteem and Repetitive Negative Thinking as Mediators of the Association Between Bullying and Disordered Eating”

Table of Contents

[Table S1. Regression Analyses Examining Age as a Predictor of Eating Disorder Symptoms, Bullying Experience, Self-Esteem, and Repetitive Negative Thinking	2](#_Toc233214170)

[Table S2. Self-Esteem and Repetitive Negative Thinking as Independent Mediators of the Association Between Bullying and Eating Disorder Symptoms	3](#_Toc233214171)

[Table S3. Self-Esteem and Repetitive Negative Thinking as Parallel Mediators of the Association Between Log-Transformed Bullying and Eating Disorder Symptoms	4](#_Toc233214172)

[Table S4. Multiple-Imputation Sensitivity Analysis of the Parallel Mediation Model	5](#_Toc233214173)

# **Table S1.** *Regression Analyses Examining Age as a Predictor of Eating Disorder Symptoms, Bullying Experience, Self-Esteem, and Repetitive Negative Thinking*

| Variable | *β* | *SE* | *t* | *p* | *R^2^* |
| --- | --- | --- | --- | --- | --- |
| Eating Disorder Symptoms | -0.067 | 0.022 | -3.11 | .002 | .004 |
| Bullying | -0.095 | 0.022 | -4.40 | < .001 | .009 |
| Repetitive Negative Thinking | -0.008 | 0.022 | -0.38 | .705 | -.0004 |
| Self-Esteem | 0.018 | 0.022 | 0.810 | .418 | -.0002 |

*Note.* *β* = standardized coefficient; *SE* = standard error; *t* = *t* statistic; *p* = *p*-value; *R^2^* = adjusted *R^2^.*

# **Table S2.** *Self-Esteem and Repetitive Negative Thinking as Independent Mediators of the Association Between Bullying and Eating Disorder Symptoms*

| **Model 1** | | | | | | | |
| --- | --- | --- | --- | --- | --- | --- | --- |
|  |  |  |  | 95% Bootstrapped CI | |  |  |
| Type | Effect | *β* | *SE* | Lower | Upper | *t* | *p* |
| Indirect | Bullying 🡪 Self-Esteem 🡪 Eating Disorder Symptoms | 0.073 | 0.008 | 0.058 | 0.089 | 8.92 | < .001 |
| a path | Bullying 🡪 Self-Esteem | -0.216 | 0.021 | -0.258 | -0.175 | -10.17 | < .001 |
| b path | Self-Esteem 🡪 Eating Disorder Symptoms | -0.335 | 0.020 | -0.375 | -0.296 | -16.73 | < .001 |
| Direct | Bullying 🡪 Eating Disorder Symptoms | 0.198 | 0.027 | 0.147 | 0.251 | 7.41 | < .001 |
| Total | Bullying 🡪 Eating Disorder Symptoms | 0.270 | 0.028 | 0.218 | 0.327 | 9.67 | < .001 |
| **Model 2** | | | | | | | |
|  |  |  |  | 95% Bootstrapped CI | |  |  |
| Type | Effect | *β* | *SE* | Lower | Upper | *t* | *p* |
| Indirect | Bullying 🡪 Repetitive Negative Thinking 🡪 Eating Disorder Symptoms | 0.055 | 0.008 | 0.040 | 0.070 | 7.18 | < .001 |
| a path | Bullying 🡪 Repetitive Negative Thinking | 0.207 | 0.021 | 0.165 | 0.249 | 9.71 | < .001 |
| b path | Repetitive Negative Thinking 🡪 Eating Disorder Symptoms | 0.264 | 0.021 | 0.224 | 0.304 | 12.88 | < .001 |
| Direct | Bullying 🡪 Eating Disorder Symptoms | 0.216 | 0.027 | 0.164 | 0.267 | 8.09 | < .001 |
| Total | Bullying 🡪 Eating Disorder Symptoms | 0.270 | 0.027 | 0.217 | 0.324 | 9.90 | < .001 |

*Note.* *β* = standardized coefficient; *SE* = standard error; CI = confidence interval; *t* = *t* statistic; *p* = *p*-value.

# **Table S3.** *Self-Esteem and Repetitive Negative Thinking as Parallel Mediators of the Association Between Log-Transformed Bullying and Eating Disorder Symptoms*

| **Self-Esteem** | | | | | | | |
| --- | --- | --- | --- | --- | --- | --- | --- |
|  |  |  |  | 95% Bootstrapped CI | |  |  |
| Type | Effect | *β* | SE | Lower | Upper | *z* | *p* |
| Indirect | Bullying 🡪 Self-Esteem 🡪 Eating Disorder Symptoms | 0.062 | 0.008 | 0.047 | 0.079 | 7.84 | < .001 |
| a path | Bullying 🡪 Self-Esteem | -0.231 | 0.020 | -0.270 | -0.192 | -11.67 | < .001 |
| b path | Self-Esteem 🡪 Eating Disorder Symptoms | -0.269 | 0.024 | -0.316 | -0.221 | -11.01 | < .001 |
| **Repetitive Negative Thinking** | | | | | | | |
|  |  |  |  | 95% Bootstrapped CI | |  |  |
| Type | Effect | *β* | SE | Lower | Upper | *z* | *p* |
| Indirect | Bullying 🡪 Repetitive Negative Thinking 🡪 Eating Disorder Symptoms | 0.025 | 0.006 | 0.013 | 0.038 | 4.04 | < .001 |
| a path | Bullying 🡪 Repetitive Negative Thinking | 0.226 | 0.022 | 0.183 | 0.269 | 10.39 | < .001 |
| b path | Repetitive Negative Thinking 🡪 Eating Disorder Symptoms | 0.111 | 0.026 | 0.061 | 0.161 | 4.35 | < .001 |
| Direct | Bullying 🡪 Eating Disorder Symptoms | 0.202 | 0.024 | 0.157 | 0.249 | 8.54 | < .001 |
| Total | Bullying 🡪 Eating Disorder Symptoms | 0.289 | 0.024 | 0.243 | 0.337 | 12.07 | < .001 |

*Note. β =* standardized coefficient; *SE =* standard error; CI = confidence interval; *z* = *z* statistic; *p = p*-value*.*

# **Table S4.** *Multiple-Imputation Sensitivity Analysis of the Parallel Mediation Model*

| **Self-Esteem** | | | | | | | |
| --- | --- | --- | --- | --- | --- | --- | --- |
|  |  |  |  | Rubin-pooled 95% CI | |  |  |
| Type | Effect | *β* | *SE* | Lower | Upper | *z* | *p* |
| Indirect | Bullying 🡪 Self-Esteem 🡪 Eating Disorder Symptoms | 0.059 | 0.007 | 0.045 | 0.073 | 8.18 | < .001 |
| a path | Bullying 🡪 Self-Esteem | -0.217 | 0.021 | -0.258 | -0.175 | -10.20 | < .001 |
| b path | Self-Esteem 🡪 Eating Disorder Symptoms | -0.270 | 0.020 | -0.310 | -0.230 | -13.30 | < .001 |
| **Repetitive Negative Thinking** | | | | | | | |
|  |  |  |  | Rubin-pooled 95% CI | |  |  |
| Type | Effect | *β* | *SE* | Lower | Upper | *z* | *p* |
| Indirect | Bullying 🡪 Repetitive Negative Thinking 🡪 Eating Disorder Symptoms | 0.024 | 0.005 | 0.015 | 0.034 | 4.96 | < .001 |
| a path | Bullying 🡪 Repetitive Negative Thinking | 0.206 | 0.021 | 0.164 | 0.248 | 9.62 | < .001 |
| b path | Repetitive Negative Thinking 🡪 Eating Disorder Symptoms | 0.117 | 0.020 | 0.078 | 0.157 | 5.80 | < .001 |
| Direct | Bullying 🡪 Eating Disorder Symptoms | 0.190 | 0.020 | 0.150 | 0.230 | 9.28 | < .001 |
| Total | Bullying 🡪 Eating Disorder Symptoms | 0.273 | 0.021 | 0.232 | 0.313 | 13.20 | < .001 |

***Note.*** Estimates were obtained using multiple imputation by chained equations with predictive mean matching (20 imputations) and pooled using Rubin’s rules. *β* = standardized coefficient; *SE =* standard error; CI = confidence interval; *z* = *z* statistic; *p = p*-value*.*
